# Associations of cumulative voriconazole dose, treatment duration, and alkaline phosphatase with voriconazole-induced periostitis

**DOI:** 10.1007/s00256-024-04707-2

**Published:** 2024-05-17

**Authors:** Walid Ashmeik, Silvia Schirò, Gabby B. Joseph, Thomas M. Link

**Affiliations:** 1 Department of Radiology and Biomedical Imaging, University of California San Francisco, 185 Berry Street, Suite 350, San Francisco, CA 94107 USA; 2 Department of Medicine and Surgery (DiMeC), University of Parma, Parma, Italy

**Keywords:** Voriconazole-induced periostitis, Fungal infection, Skeletal fluorosis, Medication-related bone disease

## Abstract

**Objective:**

To investigate the associations of cumulative voriconazole dose, treatment duration, and alkaline phosphatase with voriconazole-induced periostitis.

**Materials and methods:**

One hundred and thirty-one patients with voriconazole use were identified using a clinical informatics tool. Health record data including age, sex, immune status, alkaline phosphatase, voriconazole levels, voriconazole dose, frequency, and treatment duration were collected. Imaging studies during the duration of treatment were reviewed by two radiology trainees and imaging features of voriconazole-induced periostitis were confirmed by a board-certified musculoskeletal radiologist. The length, location in the body, location in the bone, type, and morphology of periostitis lesions were recorded. Incident voriconazole-induced periostitis was defined as new periostitis on imaging after 28 days or more of voriconazole treatment in the absence of an alternative diagnosis. Univariate Firth’s logistic regression models were performed using cumulative voriconazole dose, treatment duration, and average ALP as predictors and incident VIP as the outcome.

**Results:**

There were nine patients with voriconazole-induced periostitis and 122 patients without voriconazole-induced periostitis. The most common lesion location in the body was the ribs (37%) and morphology was solid (44%). A 31.5-g increase in cumulative voriconazole dose was associated with 8% higher odds of incident periostitis. Increased treatment duration (63 days) and higher average alkaline phosphatase (50 IU/L) were associated with 7% higher odds of periostitis and 34% higher odds of periostitis, respectively.

**Conclusion:**

Increased cumulative voriconazole dose, treatment duration, and average alkaline phosphatase were associated with higher odds of voriconazole-induced periostitis.

**Supplementary Information:**

The online version contains supplementary material available at 10.1007/s00256-024-04707-2.

## Introduction

Fungal infections are an important cause of morbidity and mortality responsible for at least 1.5 million deaths globally per year [1] and disproportionately impacting immunocompromised patients. Given its excellent bioavailability and broader spectrum of activity relative to traditional triazole agents such as itraconazole and fluconazole [2], voriconazole is commonly used for the treatment of and prophylaxis against invasive fungal infections such as invasive aspergillosis. Voriconazole’s most common side effects include visual disturbances, skin rashes, nausea and vomiting, liver toxicity, and QT prolongation [2].

Furthermore, there are several case reports of periostitis developing in patients on chronic voriconazole therapy [3–9]. Clinically, patients present with diffuse bone pain, myalgias, and elevated serum alkaline phosphatase and fluoride levels. The imaging features include multifocal periosteal reaction or thickening in the axial and appendicular skeleton, enthesopathy [10, 11], capsulitis [12], and radiotracer uptake on technetium 99 m–labeled methylene diphosphonate (MDP) bone scans [3]. This phenomenon of radiographically dramatic periostitis resolves upon discontinuation of voriconazole. Given the imaging resemblance to skeletal fluorosis and voriconazole’s tri-fluorinated molecular structure, the underlying pathophysiology is thought to relate to excess fluoride and enhanced osteoblastic activity [8, 9, 13].

Although voriconazole-induced periostitis (VIP) has been described in the literature, studies have been limited by small sample sizes and variable radiological findings between cases. The goal of this study is to investigate the associations of cumulative voriconazole dose, treatment duration, and alkaline phosphatase with VIP.

## Materials and methods

### Study patients and data collection

This retrospective study was approved by the UCSF institutional review board. A search of radiology reports was performed using a clinical informatics tool (mPower, Nuance Communications Inc, Burlington, MA) with the search terms (*voriconazole* OR *vori* OR *vfend*). One hundred and thirty-five patients with reported voriconazole use were identified. Data collected from the electronic health records included prescribed voriconazole dose, frequency, route of administration, and treatment duration. Age at voriconazole initiation, sex, immune status, alkaline phosphatase (ALP), and voriconazole levels during the duration of treatment were also collected. Patients were defined as immunocompromised if any of the following applied during voriconazole treatment: history of malignancy, solid-organ or bone marrow transplant, HIV infection, genetic immunodeficiency, diabetes mellitus, use of steroids or other immunosuppressant medications.

### Imaging and image analysis

All radiographs, computed tomography (CT), magnetic resonance imaging (MRI), bone scans, and positron emission tomography (PET) scans during the duration of treatment were reviewed by two radiology trainees (WA, SS), and imaging features of VIP were confirmed by a board-certified musculoskeletal radiologist (TL). VIP lesions were initially diagnosed and best characterized on radiographs and CT studies which were used for morphological analysis of the lesions. Standard radiographs were obtained of various bones and joints in at least two planes. CT studies of the chest were obtained on three different scanners—General Electric (GE) LightSpeed VCT, GE Discovery CT750 HD, and Phillips Brilliance 64—using the acquisition parameters listed in Supplementary Table 1.

**Fig. 1:**
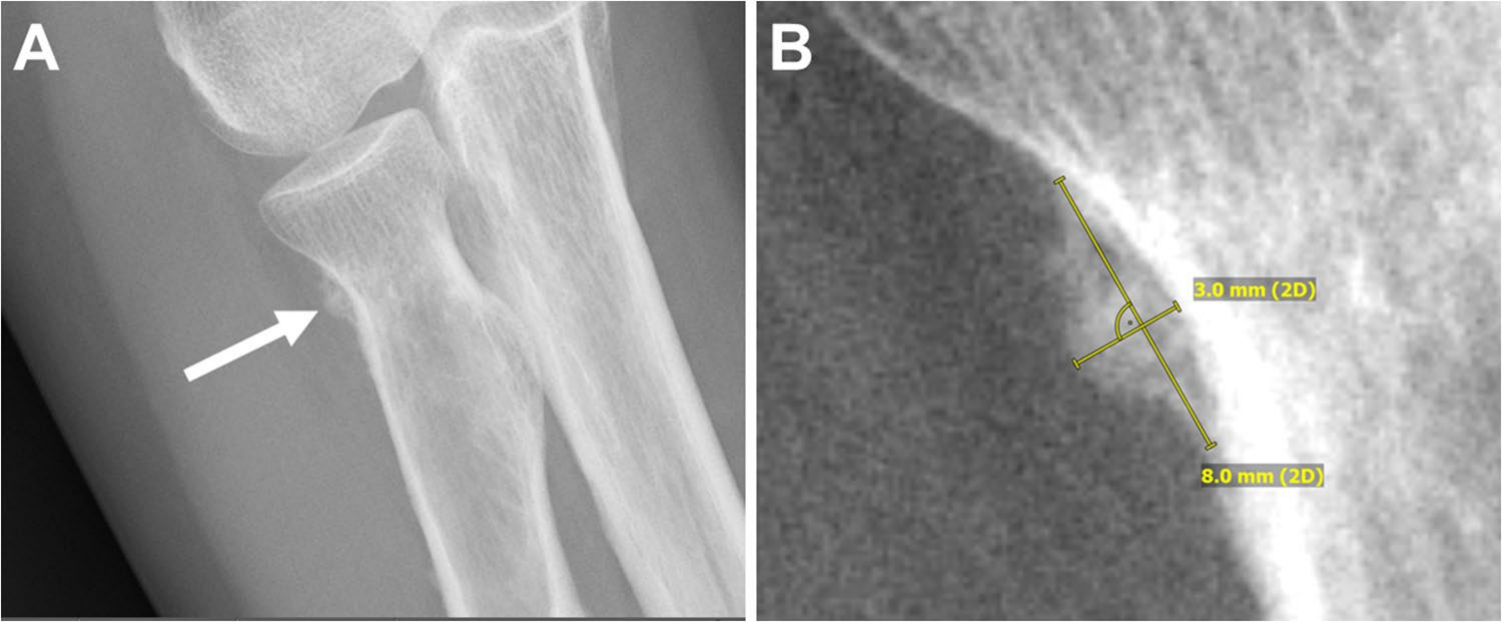
Fifty-nine-year-old woman presents with right elbow pain in the setting of chronic voriconazole use. **A** Radiograph demonstrates periostitis at the right radial neck with a continuous-type, lobulated morphology (arrow). **B** On the magnified image, this lesion measures 8 × 3 mm in length and width, respectively

**Fig. 2:**
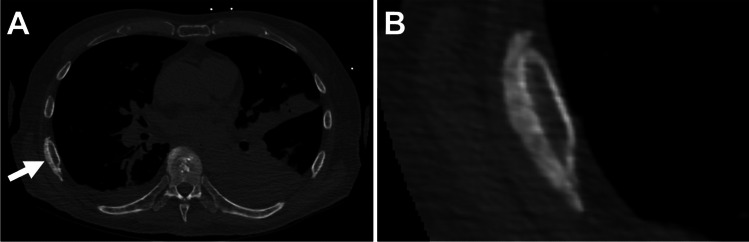
Sixty-seven-year-old man with history of polycythemia vera and allogeneic stem cell transplantation on prophylactic voriconazole therapy. **A** Axial non-contrast CT image of the chest shows periostitis at the posterolateral aspect of the right seventh rib (arrow). **B** On the magnified image, this lesion demonstrates continuous-type, solid morphology

The length, location in the body, location in the bone, type, and morphology of VIP lesions were recorded. The lesion type was classified as either continuous or interrupted. Lesions were further subclassified based on morphology as eggshell, lobulated, soap bubbles, solid, single layer, multilayered, spiculated or sunburst for continuous-type lesions and wedge-shaped, Codman triangle, interrupted onion skins, or interrupted spiculae for interrupted-type lesions [14]. Representative lesions are included in Figs. 1 and 2.

Incident VIP was defined as new periostitis on imaging after 28 days or more of voriconazole treatment in the absence of an alternative diagnosis such as bone infection, metabolic disorders, and paraneoplastic disorders. Three patients were excluded from the analysis given that voriconazole dose, frequency, and treatment duration could not be confirmed due to incomplete or missing medical records. An additional patient was excluded from the analysis because although they developed new periostitis and enthesopathy after chronic voriconazole use, it could not be confirmed that their lesions were solely secondary to voriconazole use. Cumulative voriconazole dose was calculated as the summation of the prescribed daily dose from the date of initiation until the date of incident VIP. Voriconazole treatment duration was defined as the difference between the date of prescribed voriconazole initiation and the date of incident VIP. Average ALP was calculated as the mean of ALP measurements for each patient during the duration of treatment.

### Statistical analysis

Differences in the group characteristics between patients with VIP and patients without VIP were assessed using the Mann–Whitney *U* test for continuous variables and the chi-square test or Fisher’s exact test for categorical variables as appropriate. To reduce small-sample bias in maximum likelihood estimation, univariate Firth’s logistic regression models were performed. These models used incident VIP as the outcome and cumulative voriconazole dose, treatment duration, and average ALP as predictors, with incremental changes set at 31.5 g, 63 days, and 50 IU/L, respectively. Incremental changes for cumulative voriconazole dose and treatment duration were selected based on treatment guidelines for invasive aspergillosis [15], a common indication for voriconazole use. Incremental change in ALP was selected as 50 IU/L as this may increase the ALP above the upper limit of normal and signal an alteration in bone metabolism warranting clinical attention. Predictors were selected a priori based on the literature review of VIP [7, 8, 16, 17]. Furthermore, we performed a sensitivity analysis to assess whether the results differed after adjusting for age in multivariate Firth’s logistic regression models (two predictors, one outcome). We conducted additional sensitivity analyses to assess for potential selection bias arising from the exclusion of the previously noted four patients. In these analyses, the patient presenting with lesions potentially attributable to voriconazole use was categorized alternately as both affected and unaffected by VIP in distinct sensitivity analyses. All predictors and covariates were analyzed as linear variables. Statistical analysis was performed using Stata software (Version 15, College Station, TX, USA; StataCorp LP). *P*-values < 0.05 were considered as statistically significant.

## Results

### Patient characteristics

Patient characteristics are summarized in Table 1. There were nine patients with VIP and 122 patients without VIP. Compared to patients without VIP, patients with VIP had higher average ALP (276.0 vs 129.5 IU/L, *P* = 0.002). There was insufficient evidence to conclude that the distributions of age, sex, immunocompromised status, voriconazole level, and treatment duration were significantly different between groups defined by VIP status. Additionally, there was a trend that patients with VIP had a higher cumulative voriconazole dose than those without VIP.

**Table 1:** Descriptive characteristics of patients

	No VIP (*n* = 122)	VIP (*n* = 9)	*P*	Reference range
Age, median (IQR) years	50.8 (20.2, 64.1)	60.2 (53.5, 62.0)	0.15	
Sex, *n* (%)			1.00	
Female	54 (44%)	4 (44%)		
Male	68 (56%)	5 (56%)		
Immunocompromised status, *n* (%)			0.59	
Not immunocompromised	11 (9%)	1 (11%)		
Immunocompromised	111 (91%)	8 (89%)		
Cumulative voriconazole dose, median (IQR) g	68.5 (25.0, 174.3)	96.1 (66.0, 522.5)	0.09	
Voriconazole treatment duration, median (IQR) days	167.0 (69.0, 378.0)	238.0 (132.0, 1292.0)	0.11	
Voriconazole level closest to treatment end date, median (IQR) µg/mL	2.3 (1.0, 3.3)	1.3 (0.8, 3.4)	0.59	2.0–6.0 µg/mL
Max voriconazole level during treatment, median (IQR) µg/mL	4.3 (3.0, 6.1)	4.8 (1.5, 6.4)	0.84	2.0–6.0 µg/mL
Average ALP during treatment, median (IQR) IU/L	129.5 (84.8, 171.5)	276.0 (162.0, 393.0)	0.002	38–108 IU/L

Data are given as medians (interquartile range, IQR) or *n* (% of total). Differences in the group characteristics were assessed using the Mann-Whitney *U* test for continuous variables and the chi-square test or Fisher’s exact test for categorical variables as appropriate

*VIP* voriconazole-induced periostitis, *ALP* alkaline phosphatase, *IU* international units

### Imaging characteristics

The imaging characteristics of VIP lesions are described in Table 2. All lesions were diagnosed on standard radiographs and/or CT studies. The total number of lesions was 116. The median number of lesions per patient was 10 (interquartile range of 6–16) ranging from 3 to 36 lesions per patient. The mean length was 26.5 (11.5) mm ranging from 2.8 to 112.0 mm. The most common lesion locations in the body were the ribs (37%), hands (23%), and legs (16%). The most common lesion locations in the bone were the diaphysis (42%) and body of the ribs (37%). Most of the lesions demonstrated continuous-type periosteal reactions (87%). The most common lesion morphology was solid (44%) followed by lobulated (29%) and wedge-shaped (13%). When comparing imaging modalities, there were 59 lesions diagnosed by radiographs and 57 lesions diagnosed by CT studies. The distributions for lesion type and morphology by modality are described in Supplementary Table 2. Imaging characteristics of VIP lesions by patient are included in Supplementary Table 3.

**Table 2:** Imaging characteristics of VIP lesions

	Patients with VIP (*n* = 9)
Total number of lesions, *N*	116
Number of lesions per patient, median (IQR)	10 (6–16)
Number of lesions per patient, min–max	3–36
Length of lesions, mean (SD) mm	26.5 (11.5)
Length of lesions, min–max mm	2.8–112.0
Lesion location in the body, *n* (%)	
Ribs	43 (37%)
Hands	27 (23%)
Legs	19 (16%)
Arms	15 (13%)
Scapulae	8 (7%)
Clavicles	2 (2%)
Costovertebral joints	1 (1%)
Feet	1 (1%)
Lesion location in the bone, *n* (%)	
Diaphysis	49 (42%)
Body	43 (37%)
Metaphysis	9 (8%)
Epiphysis	7 (6%)
Fossa	7 (6%)
Glenoid	1 (1%)
Lesion type, *n* (%)	
Continuous	101 (87%)
Interrupted	15 (13%)
Lesion morphology, *n* (%)	
Solid	51 (44%)
Lobulated	34 (29%)
Wedge-shaped	15 (13%)
Eggshell	6 (5%)
Multilayered	5 (4%)
Single layer	4 (3%)
Soap bubbles	1 (1%)

Data are given as medians (interquartile range, IQR) or *n* (% of total). Differences in the group characteristics were assessed using the Mann-Whitney *U* test for continuous variables and the chi-square test or Fisher’s exact test for categorical variables as appropriate

*VIP* voriconazole-induced periostitis, *ALP* alkaline phosphatase, *IU* international units

### Associations of cumulative voriconazole dose, treatment duration, and alkaline phosphatase with incident VIP

As shown in Table 3, a 31.5-g increase in cumulative voriconazole dose was associated with higher odds of incident VIP (odds ratio 1.08 [95% CI 1.02, 1.14], *P* = 0.009). Increased treatment duration (63 days) was associated with higher odds of incident VIP (odds ratio 1.07 [95% CI 1.02, 1.12], *P* = 0.005). Increased average ALP (50 IU/L) was associated with higher odds of incident VIP (odds ratio 1.34 [95% CI 1.11, 1.61], *P* = 0.002).

**Table 3:** Associations of cumulative voriconazole dose, treatment duration, and alkaline phosphatase with incident VIP

Predictors	OR	(95% CI)	*P*
Cumulative voriconazole dose	1.08	(1.02, 1.14)	0.009
Cumulative voriconazole dose^a^	1.08	(1.02, 1.14)	0.01
Treatment duration	1.07	(1.02, 1.12)	0.005
Treatment duration^a^	1.07	(1.02, 1.13)	0.006
Average ALP	1.34	(1.11, 1.61)	0.002
Average ALP^a^	1.29	(1.07, 1.54)	0.007

Data are given as odds ratios (OR) associated with increased cumulative voriconazole dose (31.5 g), treatment duration (63 days), or average ALP (50 IU/L), with 95% confidence intervals (CI)

*VIP* voriconazole-induced periostitis, *ALP* alkaline phosphatase, *IU* international units

^a^Models adjusted for age

In a sensitivity analysis adjusting for age, the associations of cumulative voriconazole dose, treatment duration, and average ALP with incident VIP remained statistically significant with estimates that were consistent in direction and similar in magnitude (Table 3). These findings remained consistent in additional sensitivity analyses evaluating for potential selection bias arising from the excluded four patients (data not shown).

## Discussion

This study investigated the associations of cumulative voriconazole dose, treatment duration, and alkaline phosphatase with incident VIP. The ribs were the most common location for VIP in the body, the diaphysis was the most common location in the bone, and the most common morphology was solid. All VIP lesions were initially diagnosed on either radiographs or CT studies and were not well characterized on MRI, bone scans, and PET scans. Higher cumulative voriconazole dose, treatment duration, and alkaline phosphatase were associated with increased odds of incident VIP. These associations remained statistically significant after adjustment for age.

VIP has been described in multiple prior case reports, case series, and observational studies [3–5, 8–12] as well as one prospective study [6]. In a retrospective study of 195 patients treated with voriconazole following an outbreak of fungal infections, Moon et al. found that patients with periostitis had higher plasma fluoride levels, daily and cumulative voriconazole dose, and serum ALP compared to those without periostitis [8]. In a different retrospective study of 43 patients with hematologic malignancies, Gerber et al. reported that bone pain and radiologic evidence of periostitis were observed in patients receiving long-term voriconazole and were not observed in the posaconazole, itraconazole, or control groups [9]. Furthermore, serum fluoride was elevated in the voriconazole group compared to the other three groups. A prospective study following ten post-transplant subjects who had received voriconazole for at least 6 months and ten post-transplant control subjects demonstrated elevated plasma fluoride levels in the group taking voriconazole [6]. Half of the subjects in the voriconazole group also had evidence of periostitis.

In our study, there were nine cases of VIP among 122 patients (7%) with the reported incidence in the literature ranging from 5 to 50% [6–9]. Similar to Moon et al. [8], our results showed that a common location in the body for VIP was the ribs and that patients with VIP had higher ALP compared to those without VIP. It is plausible to suggest that the longer a patient is on voriconazole and the higher the cumulative dose, the higher the risk of developing VIP. Furthermore, the association of increased average ALP levels with higher odds of VIP suggests that ALP might be a useful biomarker to monitor in patients on voriconazole therapy. While nonspecific, elevated ALP levels may reflect increased bone metabolism and periostitis and therefore may necessitate further diagnostic evaluation. Most of the lesions in the current study demonstrated continuous-type periosteal reactions and solid or lobulated morphologies which are seen with non-aggressive, slow processes [14]. However, a few lesions exhibited interrupted-type periosteal reactions and wedge-shaped morphology which are typically seen in more aggressive processes such as malignancy when the underlying insult outpaces the ability of the periosteum to contain it.

**Fig. 3:**
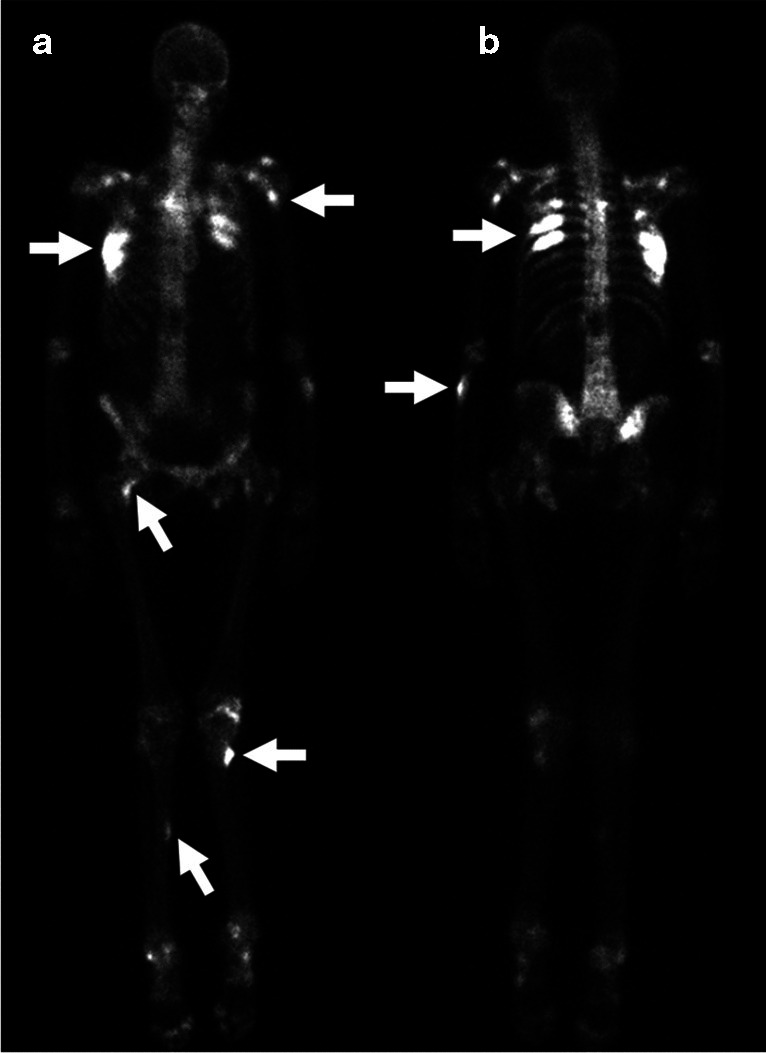
Fifty-two-year-old woman with history of liver transplant and disseminated Secdosporium infection on long-term voriconazole therapy. **A** Whole-body technetium 99 m bone scan with anterior and posterior views demonstrates multiple foci of radiotracer uptake, most pronounced in the bilateral ribs, proximal left humerus, proximal left ulna, medial right femoral neck, left proximal femoral diaphysis, and right mid femoral diaphysis

The imaging findings of VIP on MRI are varied and included nodular periostitis of the phalanges with intermediate T1 and high T2 signal [18], irregular and thick periosteal edema along the proximal femoral shafts [19], and enthesopathy and capsulitis of the glenohumeral joint [12]. Technetium 99 m–labeled MDP bone scan is sensitive for periostitis and demonstrates multifocal increased radiotracer uptake involving affected bones [4, 20, 21]. The pattern of uptake can be linear or focal and more pronounced along the metaphysis and diaphysis of the long bones [20–22]. A whole-body bone scan can identify multiple sites of periostitis and help characterize the extent of the disease in a single study (Fig. 3).

The exact pathophysiology of VIP is unknown. One of the potential mechanisms underlying VIP relates to excess fluoride, as prior studies have shown elevated plasma fluoride levels in patients with VIP [6–9, 23]. Voriconazole has three fluorine atoms and a 400-mg dose of voriconazole contains approximately 65 mg of fluoride, far exceeding the adequate daily intake of 3–4 mg outlined by the United States Department of Agriculture [6, 24]. Fluoride has a greater affinity for calcium than hydroxide ions, leading to fluorapatite replacing hydroxyapatite in the bone matrix and stimulating bone formation and periosteal reaction [25]. Voriconazole may also induce periostitis through a fluoride-independent mechanism by increasing the expression of cytokines that enhance osteoblastic activity [14]. Several studies have noted VIP developing in patients with normal and even subtherapeutic voriconazole levels [8, 9, 26]. Furthermore, individual variability in metabolism and cytochrome P450 enzymes might also play a role in susceptibility to VIP [21].

There are several study limitations that should be acknowledged. First, this is a retrospective study with a limited number of patients although our sample size was comparable to that of similar studies. Given the small number of patients with VIP, this study might be underpowered to detect small to moderate effect sizes and the results should be interpreted with caution. The retrospective nature of this study further limits our analysis of patient adherence with voriconazole intake as cumulative voriconazole dose and treatment duration are based on prescription data and are meant to represent the intended medication exposure. Moreover, plasma fluoride measurements which have been linked to VIP were not available in our study. Potential confounders including indication for voriconazole use as well as patient-specific variables such as comorbidities, variations in cytochrome P450 enzymes, the occurrence of other adverse effects (e.g. hepatotoxicity), and concurrent medications were not controlled for and may have influenced our results. The effect of time was not accounted for in our statistical analysis, and our findings, though informative, likely do not capture the entirety of the complex relationship between cumulative dose, time, and incident VIP. Furthermore, weighted cumulative exposure models may be more appropriate for long-term exposures that vary over time [27]. Finally, our findings do not establish causality, and prospective longitudinal studies are better suited for assessing causal relationships between voriconazole and periostitis.

In conclusion, this study showed that increased cumulative voriconazole dose, treatment duration, and higher average ALP were associated with higher odds of VIP. VIP is an important entity for radiologists to recognize and should be considered in patients taking voriconazole with bone pain, elevated ALP, and/or periostitis. A greater understanding of the imaging features of this condition is essential given that voriconazole discontinuation is effective at reversing the disease course.

## Supplementary Information

Below is the link to the electronic supplementary material.

Supplementary file1 (DOCX 36 KB)

## Data Availability

The data that support the findings of this study are available from the corresponding author upon reasonable request.
