# Supplementary material for: Associations of cumulative voriconazole dose, treatment duration, and alkaline phosphatase with voriconazole-induced periostitis
Source: Skeletal Radiol. 2024 May 17;54(1):41–7. doi: 10.1007/s00256-024-04707-2 (PMC11573802; doi:10.1007/s00256-024-04707-2)
Supplement: Supplementary file 1 — Supplementary file1 (DOCX 36 KB) [file 256_2024_4707_MOESM1_ESM.docx]

| **Supplementary Table 1.** CT image acquisition parameters | | | |
| --- | --- | --- | --- |
|  | GE LightSpeed VCT | GE Discovery CT750 HD | Phillips Brilliance 64 |
| Voltage (kVp) | 120 | 120 | 120 |
| Current (mA) | ATCM (reference of 120 mA) | ATCM (reference of 150 mA) | ATCM (reference of 100 mA) |
| Rotation time (s) | 0.5 | 0.5 | 0.4 |
| Pitch | 0.984 | 0.984 | 0.798 |
| Slice thickness (mm) | 1.25 | 1.25 | 3 |
| Field of view (mm^2^) | 360 | 325 | 319 |
| Reconstruction kernel | Standard | Standard | Standard |
| Abbreviations: CT = computed tomography; GE = General Electric; ATCM = automatic tube current modulation. | | | |

| **Supplementary Table 2.** Imaging characteristics of VIP lesions by modality | | |
| --- | --- | --- |
|  | CT | XR |
| Total number of lesions, N | 57 | 59 |
| Lesion type, n (%) |  |  |
| Continuous | 44 (77%) | 57 (97%) |
| Interrupted | 13 (23%) | 2 (3%) |
| Lesion morphology, n (%) |  |  |
| Solid | 43 (75%) | 8 (14%) |
| Lobulated | 1 (2%) | 33 (56%) |
| Wedge-shaped | 13 (23%) | 2 (3%) |
| Eggshell | 0 (0%) | 6 (10%) |
| Multilayered | 0 (0%) | 5 (8%) |
| Single layer | 0 (0%) | 4 (7%) |
| Soap bubbles | 0 (0%) | 1 (2%) |
| Data are given as total number (N) or n (% of total).  Abbreviations: VIP = voriconazole-induced periostitis. | | |

| **Supplementary Table 3.** Imaging characteristics of VIP lesions by patient. | | | | | | | | | |
| --- | --- | --- | --- | --- | --- | --- | --- | --- | --- |
|  | Patient 1 | Patient 2 | Patient 3 | Patient 4 | Patient 5 | Patient 6 | Patient 7 | Patient 8 | Patient 9 |
| Total number of lesions, N | 16 | 36 | 3 | 7 | 21 | 12 | 5 | 6 | 10 |
| Lesion location in the body, n (%) |  |  |  |  |  |  |  |  |  |
| Ribs | 0 (0%) | 24 (67%) | 0 (0%) | 0 (0%) | 0 (0%) | 4 (33%) | 5 (100%) | 6 (100%) | 4 (40%) |
| Hands | 4 (25%) | 0 (0%) | 0 (0%) | 1 (14%) | 17 (81%) | 0 (0%) | 0 (0%) | 0 (0%) | 5 (50%) |
| Legs | 10 (63%) | 0 (0%) | 2 (67%) | 0 (0%) | 0 (0%) | 7 (58%) | 0 (0%) | 0 (0%) | 0 (0%) |
| Arms | 2 (12%) | 3 (8%) | 0 (0%) | 6 (86%) | 4 (19%) | 0 (0%) | 0 (0%) | 0 (0%) | 0 (0%) |
| Scapulae | 0 (0%) | 6 (17%) | 0 (0%) | 0 (0%) | 0 (0%) | 1 (8%) | 0 (0%) | 0 (0%) | 1 (10%) |
| Clavicles | 0 (0%) | 2 (6%) | 0 (0%) | 0 (0%) | 0 (0%) | 0 (0%) | 0 (0%) | 0 (0%) | 0 (0%) |
| Costovertebral joints | 0 (0%) | 1 (3%) | 0 (0%) | 0 (0%) | 0 (0%) | 0 (0%) | 0 (0%) | 0 (0%) | 0 (0%) |
| Feet | 0 (0%) | 0 (0%) | 1 (33%) | 0 (0%) | 0 (0%) | 0 (0%) | 0 (0%) | 0 (0%) | 0 (0%) |
| Lesion location in the bone, n (%) |  |  |  |  |  |  |  |  |  |
| Diaphysis | 12 (75%) | 2 (5%) | 1 (33%) | 5 (71%) | 19 (90%) | 5 (42%) | 0 (0%) | 0 (0%) | 5 (50%) |
| Body | 0 (0%) | 24 (67%) | 0 (0%) | 0 (0%) | 0 (0%) | 4 (33%) | 5 (100%) | 6 (100%) | 4 (40%) |
| Metaphysis | 3 (19%) | 2 (5%) | 0 (0%) | 2 (29%) | 0 (0%) | 2 (17%) | 0 (0%) | 0 (0%) | 0 (0%) |
| Epiphysis | 1 (6%) | 2 (5%) | 2 (63%) | 0 (0%) | 2 (10%) | 0 (0%) | 0 (0%) | 0 (0%) | 0 (0%) |
| Fossa | 0 (0%) | 5 (14%) | 0 (0%) | 0 (0%) | 0 (0%) | 1 (8%) | 0 (0%) | 0 (0%) | 1 (10%) |
| Glenoid | 0 (0%) | 1 (3%) | 0 (0%) | 0 (0%) | 0 (0%) | 0 (0%) | 0 (0%) | 0 (0%) | 0 (0%) |
| Lesion type, n (%) |  |  |  |  |  |  |  |  |  |
| Continuous | 14 (88%) | 23 (64%) | 3 (100%) | 7 (100%) | 21 (100%) | 12 (100%) | 5 (100%) | 6 (100%) | 10 (100%) |
| Interrupted | 2 (12%) | 13 (36%) | 0 (0%) | 0 (0%) | 0 (0%) | 0 (0%) | 0 (0%) | 0 (0%) | 0 (0%) |
| Lesion morphology, n (%) |  |  |  |  |  |  |  |  |  |
| Solid | 7 (44%) | 23 (64%) | 1 (33%) | 0 (0%) | 0 (0%) | 5 (42%) | 5 (100%) | 5 (83%) | 5 (50%) |
| Lobulated | 4 (25%) | 0 (0%) | 2 (63%) | 2 (29%) | 18 (86%) | 2 (17%) | 0 (0%) | 1 (17%) | 5 (50%) |
| Wedge-shaped | 2 (13%) | 13 (36%) | 0 (0%) | 0 (0%) | 0 (0%) | 0 (0%) | 0 (0%) | 0 (0%) | 0 (0%) |
| Eggshell | 1 (6%) | 0 (0%) | 0 (0%) | 3 (43%) | 0 (0%) | 2 (17%) | 0 (0%) | 0 (0%) | 0 (0%) |
| Multilayered | 1 (6%) | 0 (0%) | 0 (0%) | 1 (14%) | 3 (14%) | 0 (0%) | 0 (0%) | 0 (0%) | 0 (0%) |
| Single layer | 1 (6%) | 0 (0%) | 0 (0%) | 1 (14%) | 0 (0%) | 2 (17%) | 0 (0%) | 0 (0%) | 0 (0%) |
| Soap bubbles | 0 (0%) | 0 (0%) | 0 (0%) | 0 (0%) | 0 (0%) | 1 (8%) | 0 (0%) | 0 (0%) | 0 (0%) |
| Data are given as total number (N) or n (% of total).  Abbreviations: VIP = voriconazole-induced periostitis. | | | | | | | | | |
